# Assessment of SARS-CoV-2 (COVID-19) Clinical Mouthwash Protocol and Prevalence of the Oral Pathogen *Scardovia wiggsiae*: A Pilot Study of Antibacterial Effects

**DOI:** 10.3390/mps6040065

**Published:** 2023-07-06

**Authors:** Melika Shayegh, Chase Sorenson, Jackson Downey, Summer Lin, Yuxin Jiang, Praneeti Sodhi, Victoria Sullivan, Katherine M. Howard, Karl Kingsley

**Affiliations:** 1 Department of Advanced Education in Pediatric Dentistry, School of Dental Medicine, University of Nevada, Las Vegas, 1700 W Charleston Boulevard, Las Vegas, NV 89106, USA; shayem1@unlv.nevada.edu (M.S.); sodhip1@unlv.nevada.edu (P.S.); victoria.sullivan@unlv.edu (V.S.); 2 Department of Clinical Sciences, School of Dental Medicine, University of Nevada, Las Vegas, 1700 W Charleston Boulevard, Las Vegas, NV 89106, USA; sorenc1@unlv.nevada.edu (C.S.); downej3@unlv.nevada.edu (J.D.); lins1@unlv.nevada.edu (S.L.); jiangy18@unlv.nevada.edu (Y.J.); 3 Department of Biomedical Sciences, School of Dental Medicine, University of Nevada, Las Vegas, 1001 Shadow Lane Boulevard, Las Vegas, NV 89106, USA; katherine.howard@unlv.edu

**Keywords:** SARS-CoV-2, COVID-19 protocol, mouthwash, saliva, qPCR screening, *Scardovia wiggsiae*

## Abstract

One protocol in healthcare facilities and dental offices due to the COVID-19 pandemic for reducing the amount of detectable oral SARS-CoV-2 has been gargling with mouthwash for 60 s. This protocol lasts longer than the daily routine for most patients and may have unexpected benefits in reducing oral microbes as a result. This project evaluated the prevalence of the newly identified oral pathogen *Scardovia wiggsiae* before and after this procedure to determine any measurable effects. Using an approved protocol, *n* = 36 pre-mouthwash patient samples, *n* = 36 matched post-mouthwash samples, and *n* = 36 matched recall samples were identified (total sample number *n* = 108). DNA was isolated from each sample (pre-, post-mouthwash, and recall). Screening using qPCR and validated primers revealed *n* = 10/36 or 27.8% tested positive for Scardovia among the pre-mouthwash (Sample A) isolates with *n* = 3/36 or 8.3% testing positive among the post-mouthwash (Sample B) isolates. Screening of the recall (Sample C) samples has revealed *n* = 10/36, or 27.8% once again tested positive for Scardovia, demonstrating that this pathogen was found among a significant proportion of pediatric patient samples. Moreover, the COVID-19-related procedure of requiring sustained mouth washing prior to clinical treatment appears to reduce the levels of detectable Scardovia, at least initially. However, this study found no long-term effects using this isolated protocol.

## 1. Introduction

The SARS CoV-2 (COVID-19) pandemic created a number of challenges and obstacles for safely performing healthcare visits, and more specifically dental procedures [1,2]. Although measures to contain the spread and risk from respiratory droplets, including N95 respiratory protective masks and face shields, were well known and effective methods, other modes to reduce viral load were deemed prudent and necessary [3,4,5]. One protocol widely adopted in healthcare facilities and dental offices for reducing the amount of detectable oral SARS-CoV-2 has been the introduction of extended gargling with mouthwash for 60 s prior to the start of any examination or other clinical procedures [6,7].

Several randomized controlled trials (RCT) were established early in the pandemic to test the efficacy and reliability of this protocol to reduce detectable SARS CoV-2 in saliva [8,9]. Although these trials demonstrated reductions between 90–99.9% in the amount of detectable SARS-CoV-2 in saliva among these patients, more recent systematic reviews have demonstrated greater variability in the clinical results [10,11]. It was originally thought that the active agents contained within the mouthwash, such as chlorhexidine or hydrogen peroxide, might explain this variation. However, more recent evidence suggests that this may be more directly related to the characteristics of the individual patient experience, including the length of time the mouthwash is used (15–60 s) and how active the gargle or swish may be in distributing these agents to accessible areas of the mouth [12,13,14].

This COVID-19 mouth washing protocol lasts longer than the daily routine for most patients and may have unexpected benefits in reducing oral microbes as a result [15]. For example, there is some evidence that povidone–iodine antiseptics are effective in reducing common oropharyngeal infections, including those caused by gram-positive organisms such as *Staphylococcus aureus* [16,17]. However, there is a lack of evidence regarding the effects of other oral antimicrobial agents more commonly employed in dental offices to reduce SARS-CoV-2 with the longer mouth washing protocol, such as chlorhexidine and triclosan, against other pathogenic oral bacteria.

One oral pathogen of specific interest is the gram-positive organism *Scardovia wiggsiae* (*S. wiggsiae*), which has been demonstrated to increase caries risk and drive cariogenesis in the presence or absence of *Streptococcus mutans* or *Lactobacillus* species [18,19,20]. However, the few studies that have so far evaluated antimicrobial agents against this organism have mainly focused on sugar alcohols, such as xylitol or erythritol, with no evidence to date regarding the potential effects of the newly introduced COVID-19 mouth washing protocol [21,22]. Based upon this lack of evidence, the primary objective of this project is to evaluate the prevalence of the newly identified oral pathogen S. wiggsiae before and after this procedure to determine any measurable effects.

## 2. Methods

### 2.1. Project Approval

The protocol for this study was reviewed and approved by the Institutional Review Board (IRB) and Office for the Protection of Research Subjects (OPRS) at the University of Nevada, Las Vegas (UNLV). The research-exempt protocol 1717625-1 “Retrospective analysis of microbial prevalence from DNA isolated from saliva samples originally obtained from the University of Nevada, Las Vegas (UNLV) School of Dental Medicine (SDM) pediatric and clinical population” was submitted 22 February 2021 and approved on 3 March 2021.

### 2.2. Human Subjects

The original protocol for the collection of saliva samples from the UNLV SDM clinic was approved under OPRS#1305-4466M, titled “The Prevalence of Oral Microbes in Saliva from the UNLV School of Dental Medicine Pediatric and Adult Clinical Population”. Under this protocol, saliva samples were collected from volunteer patients at the beginning of their clinic appointment. Informed Consent was collected from adult patients who chose to participate, while Pediatric Patients above the age of seven were also required to provide Pediatric Assent in addition to the Informed Consent and approval of the accompanying guardian or parent. Inclusion criteria included voluntary participation in the research study with signed Informed Consent from adult patients and signed Pediatric Assent from pediatric patients in addition to the signed Informed Consent from the appropriate parent or legal guardian. Exclusion criteria included any patient who declined to participate, any patient that did not provide Informed Consent or Pediatric Assent (if appropriate) and any patient treated outside the UNLV-SDM patient clinic. In brief, unstimulated saliva was collected in sterile collection tubes which were then labeled with a randomly generated, non-duplicated number to prevent the disclosure of any patient-specific identifying information. Demographic information including patient age, race or ethnicity, and sex were noted before transferring to a biomedical laboratory for storage and processing.

### 2.3. SARS-CoV-2 (COVID-19) Protocol

Due to the COVID-19 pandemic, new protocols and procedures were developed to reduce the amount of detectable SARS-CoV-2 in saliva and oral secretions [23,24]. This involved rinsing or gargling with mouthwash solution for 60 s prior to the start of any dental procedure or treatment. The mouthwash consisted of 0.12% chlorhexidine gluconate oral rinse, which has been used as the clinical standard mouthwash in use at UNLV-SDM. Adapting to this protocol, the saliva collection procedure used three tubes labeled with the same randomly-generated, non-duplicated number labeled A (pre-mouthwash), B (post-mouthwash), and C (recall appointment). Patients and their parents/guardians who volunteered to participate were asked to provide Informed Consent and Pediatric Assent and then were given saliva collection tube A (pre-mouthwash) for the initial collection. Following the COVID-19 mouthwash, protocol patients were then given saliva collection tube B (post-mouthwash) for the second collection. At the end of the appointment, patients were given the randomly generated number to match with saliva collection tube C (recall appointment) for the third and final collection. All samples were transferred to a biomedical laboratory for storage and processing.

### 2.4. DNA Isolation

DNA was isolated from all saliva samples with the phenol chloroform extraction method using the TRIzol reagent from Invitrogen (Waltham, MA, USA). Briefly, samples were thawed and vortexed to ensure homogeneity prior to processing. Equal volumes of saliva (500 μL) and TRIzol reagent (500 μL) were mixed in a sterile microcentrifuge tube. Chloroform (200 μL) was then added. The samples were incubated on ice for ten minutes prior to centrifugation using a refrigerated microcentrifuge from Eppendorf (Hamburg, German) at 12,000× g relative centrifugal force (RCF) for 15 min at 4 °C. Following centrifugation, the upper aqueous phase was transferred to a new sterile microcentrifuge tube and an equal volume of isopropanol was added to precipitate the DNA. Samples were centrifuged again to pellet the DNA, isopropanol was removed and the pellet was washed using ethanol. Following centrifugation for five minutes, the ethanol was removed and the pellet was resuspended using 100 μL of nuclease-free water from Fisher Scientific (Waltham, MA, USA).

### 2.5. DNA Analysis

DNA purity and concentration was determined using a NanoDrop 2000 spectrophotometer from Fisher Scientific (Waltham, MA, USA). In brief, absorbances at A260 nm and A280 nm were used to determine the sample purity, which should yield a ratio at or above 1.65 for quantitative polymerase chain reaction (qPCR) screening. In addition, the DNA concentration should be at least 100 ng to provide sufficient yield for subsequent molecular screening using qPCR.

### 2.6. qPCR Screening

Screening of DNA isolated from the saliva samples from the Pre-mouthwash (Sample A), Post-mouthwash (Sample B), and Recall appointments (Sample C) was performed with quantitative polymerase chain reaction (qPCR) and the QuantStudio qPCR system from Applied Biosciences (Waltham, MA, USA). Screening reactions were performed in triplicate using the Fast SYBR green master mix and reagent system from ThermoFisher Scientific (Waltham, MA, USA) using the manufacturer-recommended protocol with validated primers. Each reaction consisted of Absolute SYBR green master mix, nuclease-free water, validated forward and reverse primers (10 μM concentration), and 2.0 μL of sample DNA. Validated primers included:

Positive control, bacterial 16S rRNA
Forward 16S rRNA primer: 5′-ACG CGT CGA CAG AGT TTG ATC CTG GCT-3′;
Reverse 16S rRNA primer: 5′-GGG ACT ACC AGG GTA TCT AAT-3′;

*Scardovia wiggsiae* (SW) primer
SW Forward primer: 5′-GTG GAC TTT ATG AAT AAG C-3′;
SW Reverse primer: 5′-CTA CCG TTA AGC AGT AAG-3′;

### 2.7. Statistical Analysis

Demographic information was analyzed using Microsoft Excel (Redmond, WA, USA). In brief, descriptive statistics (including percentages of males and females, minorities and non-minorities) were compiled and comparisons between the saliva sample demographics for these categorical variables and the overall clinic demographics were made using Chi Square analysis. Results of qPCR screening (Scardovia-positive, Scardovia-negative) were also analyzed using Chi Square and a significance level of alpha = 0.05.

## 3. Results

The total number of patients included in this study was *n* = 36 (Table 1). The study sample included roughly equal numbers of males and females, which approximates the distribution of males and females in the patient clinic, *p* = 0.5043. Analysis of the race or ethnicity reported from the initial sample collection demonstrated that most of the samples were derived from non-White or minority patients, which also closely matches the percentages of minority patients in the overall clinic population, *p* = 0.7003. Finally, the average age of patients who volunteered saliva samples was 9.2 years, which was not significantly different from the overall average age of the pediatric clinic population of 9.0 years, *p* = 0.884.

DNA was isolated from *n* = 36 Pre-mouthwash samples, *n* = 36 matched Post-mouthwash samples and *n* = 36 matched Recall samples for a total of *n* = 108 (Table 2). These data demonstrated that DNA concentration from the first time point (T1) or Pre-mouthwash samples (Sample A) was 1141.74 ng/uL with purity measured by the absorbance ratio of A260 nm and A280 averaging 1.71, which was sufficient for qPCR screening. Analysis of the DNA from the second time point (T2) or Post-mouthwash samples (Sample B) was 883.94, which was significantly lower than the average concentration from T1 (Sample A), *p* = 0.004—although DNA purity was slightly higher, with the average A260:A280 ratio at 1.75. DNA isolated from the third time point (T3) or Recall (Sample C) averaged 1350.85, which was significantly higher than either T1 or T2, *p* = 0.036, with an average DNA purity of 1.76.

**Figure 1:**
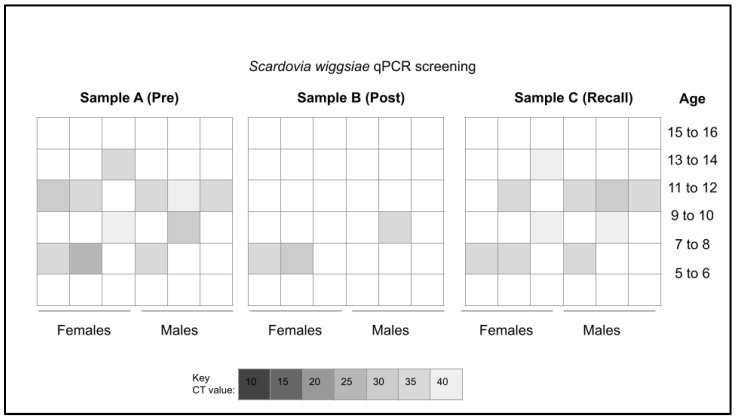
Heat map of qPCR pediatric study sample screening for *Scardovia wiggsiae* (SW). This screening revealed *n* = 10/36 or 27.8% of pre-mouthwash analysis (Sample A) harbored DNA specific for SW. The post-mouthwash analysis (Sample B) revealed only *n* = 3/36 or 8.3% tested positive for SW. Analysis of the recall or follow-up (Sample C) revealed all of the *n* = 10/36 or 27.8% tested positive for SW.

Each sample was then screened using validated primers for the gram-positive, cariogenic pathogen *Scardovia wiggsiae* or SW (Figure 1). These data demonstrated that 27.8% of the pre-mouthwash samples (Sample A) tested positive for SW, which were equally distributed between males and females. None of the samples from the youngest age range (5 to 6 years old) or the oldest age range (15 to 16 years old) tested positive for this organism. Following the COVID-19 mouthwash protocol, only three of the samples (Sample B) tested positive for SW or 8.3%, representing an overall reduction in SW-positive samples by 19.5%. Analysis of the follow-up samples demonstrated that all SW-positive samples from the pre-mouthwash (Sample A) group once again tested positive for SW in the recall appointment two days to four weeks later—with most time intervals averaging seven to ten days (Sample C).

## 4. Discussion

The primary objective of this study was to determine whether the COVID-19 mouthwash protocol had any effect on the newly identified oral pathogen *S. wiggsiae* before and after this procedure. The analysis of the results clearly demonstrated that the COVID-19 mouthwash protocol had an immediate and dramatic effect on the presence of detectable *S. wiggsiae* as measured immediately after the procedure, which may be the first clinical description of this observation in any study to date. These findings support previous literature that has suggested that other pathogenic gram-positive oral bacteria, such as *S. mutans*, may be reduced using prolonged and repeated mouth washing—although these studies were mainly among critically ill patients and those who were at risk for ventilator-associated bacterial pathogens that originate from the oral cavity [25,26].

Although these effects were temporary and all samples that were initially *Scardovia*-positive returned to *Scardovia*-positive at the recall or follow-up appointment, the fact that the microbial burden of this pathogen could be modulated by simply extending mouth washing rinse times may suggest that this protocol may have unexpected benefits for those patients who are at risk, such as children with severe early childhood caries or SECC [27,28,29]. These data are supported by other randomized controlled studies that evaluated these types of positive effects on reducing *S. mutans* using mouth washing for 60 s twice per day in children, adolescents, and high school students for periods of up to two weeks [30,31]. If there is a potential that there could be longer-term effects of this protocol on this specific pathogen, then more research should be conducted to evaluate the potential for this extended mouth-washing protocol to modulate the presence of *Scardovia* over longer periods of time [32,33].

Although many previous studies have demonstrated the effectiveness of mouthwash and mouth rinses containing chlorhexidine and fluoride against oral bacteria, these have generally been restricted to well-known oral pathogens, including *S. mutans* [34,35]. However, recent evidence has demonstrated that *S. wiggsiae* exhibits much greater fluoride tolerance than other cariogenic bacteria, compared with the more traditional cariogenic pathogens including *Lactobacillus* and *Streptococcus* species [36,37]. Given the increased attention regarding this novel cariogenic pathogen and these special characteristics that may influence oral prevalence, this information may be particularly useful to oral healthcare practitioners and researchers as they plan for and design long-term interventions that may help to modulate oral risk from this organism.

Despite the significance of these findings, there are some limitations to this study that should also be considered. For example, this was a pilot study to evaluate the effectiveness of an existing COVID-19 protocol to modulate detection and presence of another oral pathogen (not SARS-CoV-2) and therefore did not evaluate potential differences between different active ingredients within mouthwashes and mouth rinses, such as fluoride, chlorhexidine, alcohol or hydrogen peroxide [38,39]. In addition, this study also did not evaluate the potential for long-term, sustained use of prolonged mouth washing to modulate the prevalence of this organism, as was done in selected previous studies of other cariogenic pathogens [30,31]. Moreover, the time frame between the initial sample collection and recall appointments exhibited a great deal of variability, which future prospective studies might be able to control more closely. Finally, other factors, such as previous oral health and hygiene were not evaluated in this study, which may further influence the outcomes observed in this study [40,41].

## 5. Conclusions

In conjunction with the evidence that extended mouth washing can significantly reduce the presence of detectable SARS-CoV-2 in saliva from clinical patient samples, this study demonstrates that other oral bacteria including the novel cariogenic pathogen *Scardovia wiggsiae* may also be temporarily reduced either through extended exposure to the active ingredients or to the extended mechanical forces of prolonged rinsing and swishing. Although more studies will be needed to determine if this may be an effective method for reducing the oral microbial burden from this newly identified pathogen, this information is useful for researchers and oral healthcare scientists who are struggling to understand the factors that may limit the prevalence and impact of this organism among low-income and at-risk populations [42,43].

## Figures and Tables

**Table 1:** Demographic analysis of study samples.

Demographic	Study Sample	Clinic	Statistics
Sex			
Males	47.20%	52.10%	X2 = 0.446, d.f. = 1
Females	52.80%	47.90%	*p* = 0.5043
Race and Ethnicity			
White	22.20%	24.70%	X2 = 0.148, d.f. = 1
Minority	77.80%	75.30%	*p* = 0.7003
Hispanic	61.10%	52.40%	
Black	11.10%	12.20%	
Asian	2.80%	3.80%	
Age			
Average	9.16 years	9.04 years	Two tailed *t*-test, *p* = 0.884
Range	5 to 16 years	1 to 17 years	

**Table 2:** Analysis of DNA from study samples.

Study Sample	DNA Concentration	DNA Purity
		A260:A280 Ratio
Pre-mouthwash (Sample A) Time (T) 1, *n* = 36	Average: 1141.74 ng/uL +/− 38.5	Average: 1.71 Range: 1.65–1.84
	Range: 629.1–1847.3 ng/uL	
Post-mouthwash (Sample B) Time (T) 2, *n* = 36	Average: 883.94 ng/uL +/− 41.7	Average: 1.75 Range: 1.62–1.88
	Range: 663.1–1110.1 ng/uL	
	Two-tailed *t*-test T1:T2, *p* = 0.004	
Recall follow-up (Sample C) Time (T) 3, *n* = 36	Average: 1350.85 ng/uL +/− 41.7	Average: 1.76 Range: 1.61–1.85
	Range: 737.1–1207.0 ng/uL	
	Two-tailed *t*-test T1:T3, *p* = 0.036	

## Data Availability

The data presented in this study are available on request from the corresponding author.
